# RpoS-Regulated Genes and Phenotypes in the Phytopathogenic Bacterium *Pectobacterium atrosepticum*

**DOI:** 10.3390/ijms242417348

**Published:** 2023-12-11

**Authors:** Olga Petrova, Elizaveta Semenova, Olga Parfirova, Ivan Tsers, Natalia Gogoleva, Yuri Gogolev, Yevgeny Nikolaichik, Vladimir Gorshkov

**Affiliations:** 1 Kazan Institute of Biochemistry and Biophysics, Federal Research Center “Kazan Scientific Center of the Russian Academy of Sciences”, 420111 Kazan, Russia; poe60@mail.ru (O.P.); lizaolakaty@mail.ru (E.S.); parfirovaolga.i@gmail.com (O.P.); ivantsers@gmail.com (I.T.); negogoleva@gmail.com (N.G.); gogolev.yuri@gmail.com (Y.G.); 2 Institute of Fundamental Medicine and Biology, Kazan Federal University, 420008 Kazan, Russia; 3 Department of Molecular Biology, Belarusian State University, 220030 Minsk, Belarus; nikolaichik@bsu.by

**Keywords:** alternative sigma factor RpoS, bacterial stress response, cross-protection, RpoS regulon, *Pectobacterium atrosepticum*

## Abstract

The alternative sigma factor RpoS is considered to be one of the major regulators providing stress resistance and cross-protection in bacteria. In phytopathogenic bacteria, the effects of RpoS have not been analyzed with regard to cross-protection, and genes whose expression is directly or indirectly controlled by RpoS have not been determined at the whole-transcriptome level. Our study aimed to determine RpoS-regulated genes and phenotypes in the phytopathogenic bacterium *Pectobacterium atrosepticum*. Knockout of the *rpoS* gene in *P. atrosepticum* affected the long-term starvation response, cross-protection, and virulence toward plants with enhanced immune status. The whole-transcriptome profiles of the wild-type *P. atrosepticum* strain and its Δ*rpoS* mutant were compared under different experimental conditions, and functional gene groups whose expression was affected by RpoS were determined. The RpoS promoter motif was inferred within the promoter regions of the genes affected by *rpoS* deletion, and the *P. atrosepticum* RpoS regulon was predicted. Based on RpoS-controlled phenotypes, transcriptome profiles, and RpoS regulon composition, the regulatory role of RpoS in *P. atrosepticum* is discussed.

## 1. Introduction

Bacteria have considerable adaptive capacity, which enables their survival under various adverse conditions. The alternative sigma factor RpoS is often considered to be one of the major regulators that mediate alterations in gene expression in such a way as to provide an adaptive response. There are many examples showing that the knockout of the *rpoS* gene leads to a decrease in cell resistance to different stressors [1,2,3].

The adaptation of bacteria to one stress factor (primary) is known to result in the formation of so-called cross-protection, i.e., resistance to multiple (secondary) stress factors that a cell has never encountered previously [4,5]. RpoS was demonstrated to be a crucial regulator of cross-protection [6,7]; however, cases in which cross-protection occurs even if the *rpoS* gene is knocked out have also been described [8,9,10]. This indicates the existence of an RpoS-independent “side” of bacterial adaptation, whose molecular determinants, hallmarks, and mechanisms remain totally unknown to date.

RpoS is widely represented in the bacterial world and is found in distantly related species of γ-, β-, and δ-proteobacteria [11,12]. Despite RpoS conservation, the mechanisms of RpoS-dependent adaptation presumably differ significantly not only in distantly related species but also in closely related species (including those belonging to the same genus). Several facts listed below point to the variability of the mechanisms of RpoS-dependent adaptation. First, in different species, knockout of the *rpoS* gene affects the expression of different gene sets [13,14,15]. Second, RpoS regulons are represented by different genes in different species. For example, of the 186 genes attributed to the RpoS regulon of *Escherichia coli*, only 50 had orthologs in *Pseudomonas aeruginosa*, and of these 50, only 12 belonged to the RpoS regulon of *P. aeruginosa* [11]. Therefore, only 6% of genes in the RpoS regulons of *E. coli* and *P. aeruginosa* were shared by the two species. Third, even within a particular species, in two strains of *E. coli*, RpoS differentially controlled different pathways/phenotypes (tricarbolic acid cycle, flagellar motility, biofilm formation, etc.) [16,17]. Thus, despite the functional preservation of RpoS, genes within its regulons in different species/strains are likely selected in a species/strain-specific manner, enabling adaptation-related fine-tuning of metabolic processes in a species-specific way. Understanding how RpoS-mediated regulation of different gene sets in different species yields similar phenotypes (increased resistance and cross-protection) requires further in-depth experiments on a variety of different bacterial species.

RpoS and its effects were mostly investigated in animal pathogens, whereas this sigma factor has been much less studied in phytopathogenic bacteria [18]. RpoS has been shown to promote bacterial stress resistance (at least to some of the stressors) in phytopathogens such as *Ralstonia solanacearum*, *Erwinia amylovora*, and *Pectobacterium carotovorum* [19,20,21,22]. In these three species, as well as in *Dickeya dadantii* and *Pantoea ananatis*, RpoS has been demonstrated to regulate the production of different virulence factors [19,20,21,22,23,24,25,26,27,28]. In *P. ananatis* and *R. solanacearum*, RpoS positively affected the production of virulence factors [19,28], whereas, in pectolytic bacteria from the *Pectobacteriaceae* (*D. dadantii* and *P. carotovorum*), RpoS negatively regulated the production of virulence factors [20,21,24,25,27]. In *Erwinia amylovora*, RpoS differentially regulated the production of different virulence factors; herewith, RpoS effects on particular virulence factors varied in different studies, indicating that the type of RpoS-mediated regulation (positive or negative) of a specific phenotype depends on the particular test conditions [22,26]. In *Burkholderia plantarii* and *R. solanacearum*, *rpoS* deficiency was coupled with reduced virulence [19,29], whereas it was associated with increased virulence in *P. carotovorum* and *D. dadantii* [20,21,24,27]. In *Erwinia amylovora*, RpoS differentially regulated different types of plant–pathogen interactions: the *rpoS* mutant was more virulent in immature loquats, as virulent as the wild-type strain in pear plantlets, and slightly less virulent in immature pears, whereas, during incompatible interactions with tobacco plants, the *rpoS* mutant had reduced fitness compared to the wild type [22,26]. Interestingly, the hypervirulent phenotype of *P. carotovorum* appeared hypovirulent when tested on plants with reduced catalase activity (and thus an increased level of reactive oxygen species) [21]. Thus, it seems that in pectolytic bacteria, RpoS provides a shift of priority from virulence toward stress resistance.

In phytopathogenic bacteria, the effects of RpoS have not been analyzed with regard to cross-protection, except that RpoS has been shown to be necessary for full stationary-phase cross-protection (when cell resistance increases following the entrance to the stationary growth phase compared to logarithmic phase cells) in *R. solanacearum* [22]. Genes whose expression is directly or indirectly controlled by RpoS have not been determined at the whole-transcriptome level in any phytopathogenic species. This presents an obstacle to understanding the regulatory roles of RpoS in phytopathogenic bacteria. Therefore, our study aimed to determine RpoS-regulated genes and phenotypes in *Pectobacterium atrosepticum*.

## 2. Results and Discussion

### 2.1. Phenotype of the P. atrosepticum RpoS Mutant

#### 2.1.1. Growth and Primary Stress (Starvation)

**Figure 1:**
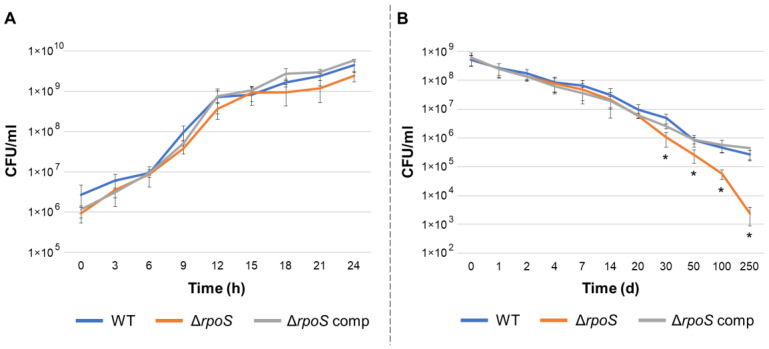
The dynamics of the colony-forming unit (CFU) titer in growing (**A**) and starving (**B**) cultures of wild-type *Pectobacterium atrosepticum* (WT, blue lines), *P. atrosepticum* Δ*rpoS* mutant (Δ*rpoS*, orange lines), and complemented Δ*rpoS* mutant carrying the *rpoS* gene within the recombinant plasmid (Δ*rpoS* comp, gray lines). The presented values are the means ± standard deviation of four-five biological replicates. Asterisks (*) show the significance of the difference between the mean values for the wild type and mutant at each time point (Mann–Whitney two-sided test, *p* < 0.05).

The growth curves of the wild type (WT), Δ*rpoS* mutant, and complemented Δ*rpoS* mutant (carrying the *rpoS* gene within the recombinant plasmid) did not differ during culturing in LB medium (Figure 1). During starvation, the colony-forming unit (CFU) titer of all three strains gradually decreased, which is typical of cultures starving at a high initial population density [30,31] (Figure 1). During the first 20 days of starvation, the CFU titer did not differ between the WT, mutant, and complemented mutant cultures. However, after 30 days of starvation, the CFU titer of mutant strain cultures was significantly lower than that of the WT and the complemented mutant (Figure 1). By the 250th day of starvation, the CFU titer in the mutant strain cultures was more than 100 times lower than that of the WT. Thus, *rpoS* is unlikely to make a significant contribution to adaptation to a rather short-term starvation of *P. atrosepticum*; however, this gene is evidently required to maintain *P. atrosepticum* fitness under long-term starvation. Previously, for bacterial species other than *P. atrosepticum*, the effect of the *rpoS* gene knockout has also been shown to be associated with the reduction in the cell titer under starvation; herewith, the observed effect was manifested both following short-term and long-term starvation, depending on the particular study [32,33,34,35,36,37].

#### 2.1.2. Cross-Protection

To assess the effect of the *rpoS* gene on the formation of cross-protection, a phenomenon of acquiring increased resistance to secondary stress factors after adaptation to the primary stress factor [6,7], we compared the resistance of the early stationary phase and starving cells of the WT, Δ*rpoS* mutant, and complemented Δ*rpoS* mutant. We have previously shown that the maximal level of cross-protection in WT *P. atrosepticum* is achieved after one day of starvation [38], and therefore, in the current experiments, one-day starving cultures were used.

**Figure 2:**
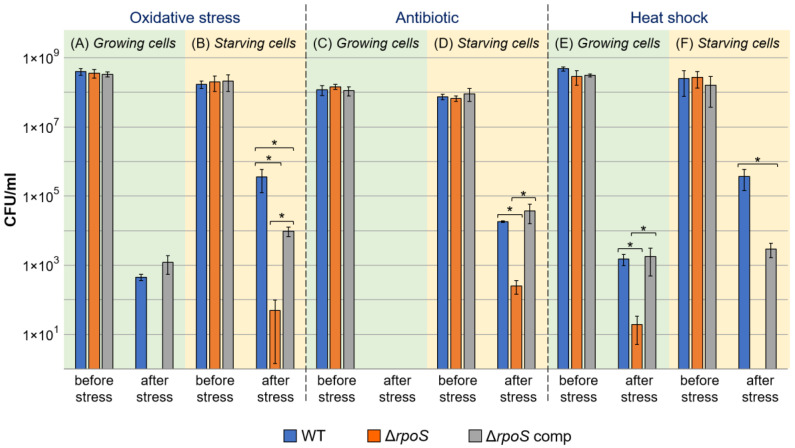
Cross-protective effect in wild-type *Pectobacterium atrosepticum* (WT, blue columns), *P. atrosepticum* Δ*rpoS* mutant (Δ*rpoS*, orange columns), and complemented Δ*rpoS* mutant carrying the *rpoS* gene within the recombinant plasmid (Δ*rpoS* comp, gray columns). The early stationary-phase (**A**,**C**,**E**) or one-day starving (**B**,**D**,**F**) cells were subjected to secondary stressors: 2.5 mM hydrogen peroxide (**A**,**B**), 40 μg/mL rifampin (**C**,**D**), or heat shock (48 °C, 5 min) (**E**,**F**). Cell titers before and after exposure to secondary stressors are presented. The presented values are the means ± standard deviation of three biological replicates. Asterisks with brackets show the significance of the difference in the mean values between the specified experimental groups (Mann–Whitney two-sided test with Bonferroni correction for multiple comparisons, *p* < 0.05).

Starving WT cells acquired cross-protection, as evident from the fact that starving WT cells were more resistant to secondary stressors (hydrogen peroxide, antibiotic rifampin, and heat shock) than stationary phase WT cells (Figure 2). The starving cells of the Δ*rpoS* mutant were much less resistant to all three secondary stressors than the starving cells of the WT. However, the formation of cross-protection to some extent was also observed for the mutant strain since starving mutant cells were more resistant than stationary phase mutant cells to two (hydrogen peroxide and rifampin) of the three secondary stressors (Figure 2). The starving cells of the complemented mutant were more resistant to all three secondary stressors than the starving cells of the mutant strain. However, the restoration of the cross-protection effect toward two of the three stressors (except rifampin) in the complemented mutant was only partial (to the level below the cross-protection in the WT) (Figure 2).

Thus, *rpoS* makes a significant contribution to the formation of cross-protection in *P. atrosepticum*, which is consistent with the demonstrated role of RpoS in cross-protection in other bacteria [6,7,39]. However, even in the absence of this gene, some level of cross-protection can be achieved in *P. atrosepticum*, pointing to the *rpoS*-independent “side” of bacterial adaptation. This is in accordance with a study performed previously on *Pseudomonas putida*, whose *rpoS*-deficient mutant displayed reduced cross-protection after starvation compared to the wild type; however, some level of cross-protection (although lower than that in the wild type) was achieved in *P. putida* even in the absence of the functional *rpoS* gene [33].

#### 2.1.3. Virulence

**Figure 3:**
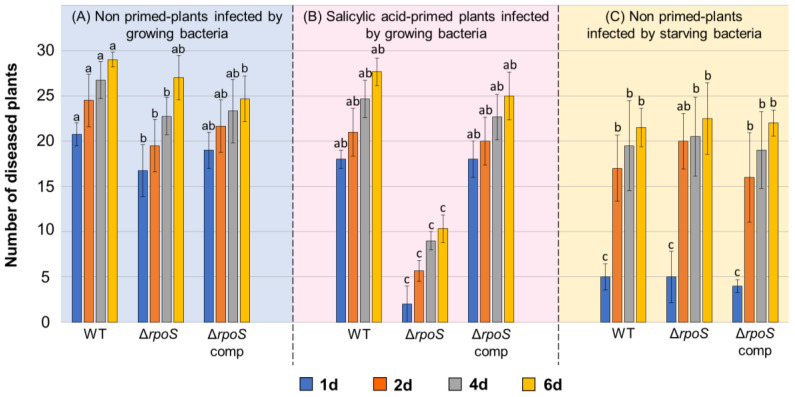
The disease incidence rate caused by the wild-type *Pectobacterium atrosepticum* (WT), *P. atrosepticum* Δ*rpoS* mutant (Δ*rpoS*), and complemented Δ*rpoS* mutant carrying the *rpoS* gene within the recombinant plasmid (Δ*rpoS* comp). Tobacco plants were infected by early stationary-phase (**A**,**B**) or one-day starving (**C**) cells. Plants were not treated (**A**,**C**) or treated (primed) with 0.2 mM salicylic acid (**B**). The disease incidence rate was assessed 1 (blue columns), 2 (orange columns), 4 (gray columns), and 6 (yellow columns) days after plant inoculation. The presented values are the means ± standard deviation of three independent experiments; within each experiment, 30 plants from each experimental group were infected. Columns corresponding to a particular time point (either 1 (blue), 2 (orange), 4 (gray), or 6 (yellow) days post inoculation) that do not share the same letter have significantly different mean values in pairwise comparison (Mann–Whitney two-sided test with Bonferroni correction for multiple comparisons, *p* < 0.05). Note that the presented statistical analysis does not consider the pairwise comparison of experimental variants at different time points.

To check the effect of *rpoS* gene knockout on *P. atrosepticum* virulence, tobacco plants were infected by the WT, Δ*rpoS* mutant, and complemented Δ*rpoS* mutant, and disease incidence was scored 1, 2, 4, and 6 days after infection. On the 1st and 6th days after infection with the WT, 69% and 96% of plants, respectively, displayed obvious disease symptoms (Figure 3). Infection by the Δ*rpoS* mutant led to statistically significantly fewer disease incidences than the WT, but the observed difference in virulence between the WT and mutant (although statistically significant) was very small and expressed only during the first four days after infection. On the 6th day after infection, the number of disease incidents did not differ between plants infected by the WT and mutant strains (Figure 3). This means that the *rpoS* mutation has only a small effect on virulence related to the dynamics of disease progression but not on the total number of disease incidences.

Since the Δ*rpoS* mutant displayed a reduced level of stress resistance compared to the WT (Figure 2), we checked the effect of *rpoS* gene knockout on *P. atrosepticum* virulence toward primed plants (in which the defense responses were induced to some extent). For plant priming, we used 0.2 mM salicylic acid (SA) since this phytohormone determines plant resistance to *P. atrosepticum*, whereas its low (0.2 mM) concentration provides resistance toward only those *P. atrosepticum* strains that are defective in stress resistance but not to the WT strain [40,41]. In our experiments, 0.2 mM SA also did not decrease the disease incidence rate in plants infected by the WT compared to WT-infected non-primed plants (Figure 3). However, at this concentration, SA strongly reduced the disease incidence caused by the Δ*rpoS* mutant. The complementation of the Δ*rpoS* mutant strain with a plasmid with a functional *rpoS* gene restored virulence toward SA-primed plants to the WT level. Thus, although *rpoS* makes only a small contribution to *P. atrosepticum* virulence toward non-primed plants, this gene is required for *P. atrosepticum* to manifest its virulence toward primed plants with enhanced immune responses.

Previous studies have shown that the effect of RpoS on bacterial virulence differs depending on the species. RpoS was demonstrated to be required for full virulence in the animal pathogens *Salmonella typhimurium*, *Vibrio cholerae*, *Vibrio anguillarum*, and *Citrobacter rodentium* [2,42,43,44]. The null *rpoS* mutation had only a small positive effect on the adherence of *Escherichia coli* to epithelial cells [45], whereas it had no effect on the virulence of *Yersinia enterocolitica* [46]. In contrast, in *Pseudonomas aeroginosa*, the *rpoS* mutation enhanced the formation of biofilms and the production of virulence factors, as well as increased virulence [37,47].

As for the plant pathogenic bacteria, the effect of RpoS on virulence was demonstrated for *Pectobacterium carotovorum* (formerly *Erwinia carotovora*), belonging to the same genus as *P. atrosepticum*. The *P. carotovorum* Δ*rpoS* mutant produced more virulence factors and was more virulent than the wild-type strain [20,21,23]. However, the virulence of the *P. carotovorum* Δ*rpoS* mutant was decreased compared with that of the wild-type strain toward plants with reduced catalase activity (and thus an increased level of reactive oxygen species) [21]. Thus, our data obtained using SA-primed plants (Figure 3) are in accordance with the results obtained for *P. carotovorum* [21] and support the conclusion that RpoS contributes to *Pectobacterium* virulence only when the pre-infection immune status of the host plant is in the primed state.

One of the important “tasks” of the bacterial stress adaptation process is to maintain pathogen virulence under unfavorable conditions. Our results showed that *rpoS* gene knockout reduced cross-protection in *P. atrosepticum* (Figure 2). Therefore, we analyzed whether a reduced level of cross-protection affected the virulence of starving cells. Starving cells of the WT (although retaining rather high virulence) were slightly less virulent than stationary phase cells of the WT (Figure 3). Herewith, no differences were observed between the virulence of starving cells of the three assayed strains (WT, Δ*rpoS* mutant, and complemented Δ*rpoS* mutant) at any of the assessed time points (Figure 3). Thus, the inability of starving *P. atrosepticum* cells to fully acquire cross-protection during stress exposure does not affect the virulence of stressed cells. This presumably means that the mechanisms of acquiring cross-protection significantly differ from those that maintain virulence under unfavorable conditions.

### 2.2. Comparative Transcriptomics of WT and ΔrpoS Mutant under Growth and Starvation Conditions

Below, we discuss gene expression profiles in four variants of comparison: (1) WT under starvation compared to WT during growth (WT_S/WT_G); (2) mutant under starvation compared to mutant during growth (Mu_S/Mu_G); (3) mutant under starvation compared to WT under starvation (Mu_S/WT_S); (4) mutant during growth compared to WT during growth (Mu_G/WT_G). The numbers of up- and downregulated genes for each comparison are provided in Table 1.

#### 2.2.1. Stress Genes

Under growth conditions, 25 genes from the supercategory “Stress” were DEGs in Mu_G/WT_G (15 down- and 10 upregulated) (Table S1). Fifteen downregulated genes (and thus positively regulated by RpoS) included six genes related to Antibiotic/Multidrug resistance, four genes related to Antioxidant systems, four genes encoding stress proteins (carbon starvation protein CstA (ECA1961), entericidin A/B family lipoprotein (ECA3975), universal stress protein UspB (ECA0049), and heat-shock chaperone IbpB (ECA4402)), and one gene related to the toxin–antitoxin system (ECA3410). Of these 15 RpoS-upregulated genes, nine were downregulated and five were non-DEGs in the WT under starvation (WT_S/WT_G) (Table S1). This means that genes that were RpoS-upregulated under growth conditions were not induced under starvation of the WT (WT_S/WT_G), indicating that the increase in the expression of these genes is unlikely to play a significant role in *P. atrosepticum* adaptation to starvation. The exception was the gene related to the toxin–antitoxin system (ECA3410), which was downregulated in Mu_G/WT_G and simultaneously upregulated in WT_S/WT_G.

Among the 10 upregulated genes in Mu_G/WT_G (and thus negatively regulated by RpoS) were three genes related to the toxin–antitoxin system, five genes related to Antibiotic/Multidrug resistance, and two genes encoding stress proteins (paraquat-inducible protein A (ECA1566) and cold-shock protein CspD (ECA2659)) (Table S1). Five of these ten RpoS-downregulated genes were upregulated (and none were downregulated) in the WT under starvation (WT_S/WT_G), indicating that despite the negative effect of RpoS on their expression, the upregulation of these genes is a characteristic of the starvation response.

Under starvation conditions, 46 genes from the supercategory “Stress” were DEGs in Mu_S/WT_S (17 down- and 29 upregulated) (Table S1). Among the 17 downregulated genes (and thus positively regulated by RpoS) were genes related to Antioxidant systems (6 genes), Stress proteins (6 genes), toxin–antitoxin system (2 genes), Degradation of xenobiotics (2 genes), and Antibiotic/Multidrug resistance (1 gene) (Table S1). Except for the one gene encoding the envelope stress response membrane protein PspB, none of the six Mu_S/WT_S-downregulated genes from the category “Stress proteins” were upregulated in WT_S/WT_G, indicating that despite positive RpoS regulation, the starvation response of the WT is not associated with the upregulation of these genes.

Twenty-nine stress-related genes (supercategory “Stress”) upregulated in Mu_S/WT_S (and thus negatively regulated by RpoS) belonged to the following categories: Antioxidant systems (10 genes), Stress proteins (9 genes), Antibiotic/Multidrug resistance (6 genes), toxin–antitoxin system (2 genes), CRISPR/Cas system (1 gene), Degradation of xenobiotics (1 gene). Of the nine genes from the category “Stress proteins” that were upregulated in Mu_S/WT_S (Table 2), six encode proteins that have been previously shown to be associated with stress responses in bacteria other than *P. atrosepticum*.

The DNA starvation/stationary phase protection protein Dps (ECA2767 in Table 2), whose gene was upregulated in both Mu_S/WT_S and Mu_S/Mu_G, is known to protect cells from oxidative stress, UV and gamma irradiation, iron and copper toxicity, thermal stress, and acid and base shock [48,49]. Dps is a DNA-binding protein that protects DNA [50,51]. Importantly, the protective effect of Dps in *E. coli* has been demonstrated to be achieved independently of RpoS [52,53]. The toxin YoeB (ECA0443 in Table 2), by promoting mRNA degradation, presumably contributes to growth termination under stress conditions in *Streptococcus pneumoniae* and *E. coli* [54,55]. RcsF (ECA3527 in Table 2) is a component of the Rcs (regulator of capsule synthesis) system that senses defects in the outer membrane and coordinates the responses directed to the neutralization of these defects [56,57]. CspD (ECA2659 in Table 2), a cold-shock protein, participates in the formation of biofilms and persisters in *E. coli,* as well as the stress resistance and virulence of *Listeria monocytogenes* [58,59,60,61]. IbpA/B (ECA4403, ECA4402 in Table 2), heat-shock proteins that form functional heterodimers, prevent the aggregation of denatured proteins, and facilitate their refolding [62].

The role of RpoS in the regulation of the production of these proteins, except for Dps [52], has not been previously discussed. Our results show that RpoS negatively regulates the expression of genes encoding these proteins in *P. atrosepticum* under starvation. Six of the nine upregulated in Mu_S/WT_S genes from the category “Stress proteins” were also upregulated during the starvation of the mutant strain (Mu_S/Mu_G) (including YoeB, ibpA/B, Dps), whereas only one of them (YoeB, ECA0443) was upregulated during the starvation of the WT (WT_S/WT_G) (Table 2). This presumably means that although the increase in the level of transcripts of these genes is not typical of the “normal” starvation response (in the WT), the synthesis of these proteins is likely to be enhanced during starvation in the mutant strain. On the one hand, although these stress-related genes were upregulated in the mutant compared to the WT, the mutant strain was more susceptible to stress factors than the WT (described above, Figure 2). On the other hand, it cannot be excluded that a possible increased production of these proteins might promote at least a low level of cross-protection in the mutant strain, partially compensating for the absence of a functional *rpoS* gene. Therefore, these proteins can be considered candidates that provide at least a partial level of stress resistance in the absence of the *rpoS* gene.

Only five genes from the supercategory “Stress” were similarly regulated under growth and starvation conditions in the mutant vs. WT (Table 3). Thus, RpoS seems to have the most pronounced impact on the regulation of the expression of these genes compared to other stress-related genes. Herein, three genes were positively regulated by RpoS (downregulated in the mutant vs. WT), and two genes were negatively regulated by RpoS (upregulated in the mutant vs. WT).

Among the genes positively regulated by RpoS under both conditions (downregulated in the mutant vs. WT) were those encoding superoxide dismutase (SodC), a well-known antioxidant enzyme involved in the detoxification of reactive oxygen species (ROS) [63,64,65], and the universal stress protein UspB involved in the formation of cell resistance [66]. However, *sodC* and *uspB* genes positively regulated by RpoS in *P. atrosepticum* were not upregulated in the WT under starvation (WT_S/WT_G), indicating that the starvation response associated with the formation of cross-protection is not coupled with the increase in the transcript levels of these genes.

RpoS is often considered from the perspective of its positive regulatory role in the expression of stress-related genes, including those encoding antioxidant proteins/enzymes, universal stress proteins, and proteins providing resistance to hyperosmotic conditions, ethanol, heat, UV, and non-optimal pH [16,66,67,68,69,70]. However, in our experiments, more stress-related genes were negatively regulated by RpoS than were positively regulated. Herewith, the positively RpoS-regulated *P. atrosepticum* genes were mostly not upregulated in the WT during starvation (WT_S/WT_G), so their upregulation by RpoS does not seem to reflect a “conventional” starvation response implemented by the WT strain. Thus, based on the characterized expression profile of the stress-related genes, RpoS can hardly be regarded as a regulator that diverts resources from different metabolic pathways to produce enhanced amounts of a variety of stress proteins that increase *P. atrosepticum* cell stress resistance.

#### 2.2.2. Transcription Factors

Under growth conditions, only 17 of 354 genes encoding transcription factors (TFs) (including sigma factors) were DEGs in Mu_G/WT_G (4 down- and 13 upregulated) (Table S1). Under starvation, 54 genes encoding TFs were DEGs in Mu_S/WT_S. Thirty TF-encoding genes were downregulated in Mu_S/WT_S. None of these 30 genes were regulated in the mutant vs. WT during growth (Mu_G/WT_G), indicating that the positive effect of RpoS on the expression of these genes is manifested only during starvation (Table S1). Among these 30 genes, more than half (17) were upregulated in WT_S/WT_G, indicating that the upregulation of these genes is typical of the starvation response of the WT, and the absence of the *rpoS* gene reduces the transcript level of these TF-encoding genes during starvation (Table 4). Thus, these 17 TFs can be regarded as candidates for further investigation from the viewpoint of their potential role in mediating the effect of RpoS and acting downstream of RpoS to promote adaptation and the formation of cross-protection.

Twenty-four TF-encoding genes were upregulated in Mu_S/WT_S (Table S1). Eight of these twenty-four genes were also upregulated in WT_S/WT_G, indicating that the corresponding TFs presumably participate in adaptation (Table 5). Thus, it can be hypothesized that the increased expression of these genes in the mutant vs. WT under starvation may confer at least a low level of stress resistance (described above) in cells lacking RpoS, partially compensating for the absence of the *rpoS* gene. The fact that *rpoS*-dependent genes can be regulated by other transcription factors in the absence of RpoS has been previously reported. For example, the primary sigma factor RpoD has a high affinity for RpoS promoters and can functionally substitute RpoS in regulating gene expression [71]. The transcription factor DksA and cAMP sensor CRP were also shown to regulate the expression of RpoS-regulated genes [72,73,74,75,76].

TF-encoding genes that were downregulated in the mutant vs. WT during both growth and starvation were not revealed. In turn, two TF-encoding genes (GntR-family transcriptional regulator (ECA0348) and putative transcriptional regulator (ECA4051)) were simultaneously upregulated under both conditions in the mutant vs. WT (Table S1), indicating that RpoS has an obvious negative effect on the expression of these genes. Both of these TF-encoding genes were non-DEGs in the WT during starvation (WT_S/WT_G) (Table S1), indicating that these TFs are unlikely to have a prominent role in stress response.

Thus, in *P. atrosepticum*, RpoS affects the expression of many genes encoding different transcription factors, some of which presumably mediate the regulation of gene expression downstream of RpoS under starvation. More than half of the TF-encoding genes positively regulated by RpoS under starvation are upregulated during the “normal” starvation response (in the WT); herewith, only one-third of the TF-encoding genes negatively regulated by RpoS under starvation are upregulated during starvation in the WT (Table S1). This presumably means that, to some extent, RpoS promotes a “shift in priority” toward the synthesis of those TF that are activated under stress conditions. However, such a “shift” is not unalloyed since some TF-encoding genes upregulated during starvation appeared negatively regulated by RpoS, whereas some TF-encoding genes positively regulated by RpoS were non-regulated or downregulated under starvation.

#### 2.2.3. Virulence

Plant-cell-wall-degrading enzymes (PCWDEs), mostly pectate lyases, are considered the major virulence factors of *Pectobacterium* species, and RpoS has been shown to negatively regulate the production of these enzymes [20,23,24,25,27]. In our experiments, of the thirty-seven annotated PCWDE-related genes (category PCWDE), only four and five genes were upregulated in the mutant vs. WT under starvation and growth conditions, respectively (Table S1). Herein, genes encoding major extracellular *P. atrosepticum* pectate lyases (PelA (ECA4067), PelB (ECA4068), and PelI (ECA1094)) were negatively regulated by RpoS under growth conditions, and one of them (PelB) was also negatively regulated under starvation conditions (Table S1). Thus, our results are in agreement with those of previous studies [20,23,24,25,27] regarding the negative effect of RpoS on the expression of pectate lyase-encoding genes.

We noted that the pectate lyase genes *pelA*, *pelB*, and *pelI* (ECA4067, ECA4068, and ECA1095) were downregulated under starvation in both the mutant and WT, whereas the *pehA* gene (ECA1095) encoding the major endopolygalacturonase was upregulated under starvation in both strains (Table S1). *pelI* and *pehA* are divergently transcribed from the shared regulatory region. We have recently shown that the PhoPQ two-component system is responsible for the antiphase expression of *pelI* and *pehA* and that this regulatory mode is involved in switching from the early to late infection stage in *P. versatile* [77]. Since *phoP* and *phoQ* expression levels were increased in the WT under starvation (Table S1), this two-component system is likely responsible for the observed difference in *pelA* and *pehA* expression in the WT cultures. However, the expression of both *pehA* and *pelI* was increased in the mutant compared to WT, and neither *phoP* nor *phoQ* expression was altered due to *rpoS* gene knockout (Table S1). Therefore, RpoS is unlikely to play a direct or PhoPQ-mediated role in switching the spectrum of polygalacturonic acid-depolymerizing enzymes in *P. atrosepticum*.

RpoS deficiency did not affect the expression levels of genes related to the Tat and Sec pathways; the type I secretion system (T1SS), T2SS, or T4SS; or siderophore-related genes (Table S1). Under growth (but not starvation) conditions, some genes related to the T3SS and T5SS were upregulated in the mutant vs. WT, pointing to some negative effects of RpoS on these secretion systems (Table S1). In turn, RpoS had a pronounced negative effect on the expression of T6SS-related genes: 26 of 42 T6SS-related genes were upregulated (and none were downregulated) in Mu_G/WT_G (Table S1). The T6SS is a well-known bacterial weapon for interspecific competition, and in some species, including *P. atrosepticum*, it is also required for virulence [78,79]. Moreover, the T6SS has also been shown to be involved in stress response in *Vibrio anguillarum* [80] and *Yersinia pseudotuberculosis* [81,82], while T6SS-related genes were shown to be positively regulated by RpoS [83]. However, in *P. atrosepticum*, we observed an evident negative effect of RpoS on T6SS-related genes.

The most pronounced effect of RpoS on the expression of virulence-related genes was observed for the gene cluster related to the synthesis of the phytotoxin coronafacic acid (cfa), a virulence factor that determines the aggressive behavior of *P. atrosepticum* [41,84,85]. All 11 genes related to cfa biosynthesis were strongly (more than 10-times) upregulated in the mutant vs. WT under growth conditions (Mu_G/WT_G), and 7 of them were also upregulated under starvation (Mu_S/WT_S) (Table S1). This likely means that RpoS strongly represses the production of this virulence factor in *P. atrosepticum*. To the best of our knowledge, the effect of RpoS on phytotoxin production in phytopathogens has not been previously discussed. However, in the animal pathogen *Vibrio cholera*, RpoS has been shown to negatively regulate the production of the cholera toxin [86].

RpoS also displayed a pronounced negative effect on the expression of flagellum-based motility-related genes. A total of 47 of the 85 genes related to flagellar assembly and chemotaxis were upregulated, and none were downregulated in the mutant vs. WT under starvation (Mu_S/WT_S) (Table S1). Under growth conditions, this RpoS effect was also evident with no downregulated genes and 20 upregulated genes in Mu_G/WT_G (Table S1). As a result, the downregulation of motility-related gene expression that occurs during the starvation of *P. atrosepticum* (this study; [38]) was much less manifested in the absence of a functional *rpoS* gene.

The obtained results on the expression of the flagellar motility-related genes were verified by us using a swimming motility assay: the macrocolonies formed by the Δ*rpoS* mutant in the semi-solid agar were significantly (Mann–Whitney two-sided test, *p* < 0.05) larger (27.6 ± 0.54 mm) than those of the WT (20.2 ± 1.79 mm) and complemented Δ*rpoS* mutant (21.8 ± 0.83 mm). This is in accordance with the negative effect of RpoS on the expression of flagellar- and chemotaxis-related genes in *E. coli* [16,87,88] as well as with the hyperflagellar phenotype of the *rpoS* mutant of *E. coli* [89]. Thus, the RpoS effect seems to determine the priority of sessile behavior over motile behavior in different bacterial species. However, a positive effect of RpoS on flagellum-based motility has also been reported: the Δ*rpoS* mutant of *Yersinia pseudotuberculosis* was characterized by reduced motility compared to the wild type [83].

Thus, our results show that among *P. atrosepticum* virulence genes, RpoS has the most pronounced effect on the expression of genes related to the T6SS, flagellar motility, and coronafacic acid synthesis. Herewith, RpoS decreases the transcript levels of these genes.

#### 2.2.4. General Metabolism and Transporters

Differences in the expression profile between the mutant and WT (mostly under starvation) were also associated with changes in the transcript levels of genes related to general metabolism (Table S1). Genes in some categories related to protein metabolism were differentially regulated in the mutant vs. WT during starvation. Some genes from the category “Amino acid metabolism” were DEGs in Mu_S/WT_S. The most pronounced effect was observed for the genes in the subcategory “Cysteine and methionine metabolism”, in which 11 of 36 genes were upregulated in the mutant vs. WT (Table S1). Four genes related to arginine biosynthesis were also upregulated in the mutant (Table S1). Many genes upregulated in the mutant vs. WT were related to the synthesis and modification of proteins, which was particularly pronounced for genes encoding ribosomal proteins. Twenty-four genes encoding ribosomal proteins were upregulated in the mutant vs. WT, and twenty-one of these genes were downregulated in the WT under starvation (WT_S/WT_G) (Table S1), indicating that the reduction in the transcript levels of these genes is typical of the starvation response. Taken together, the transcriptome profiles highlight the negative mode of RpoS control over certain aspects of protein and amino acid metabolism under starvation.

The entire cluster of genes encoding subunits of NADH-quinone oxidoreductase (ECA3016-ECA3028) (Table S1), the primary acceptor of electrons in the electron transport chain, was upregulated in the mutant vs. WT. Presumably, the increased level of the primary acceptor of electrons can create some imbalance in the electron transport chain in the mutant strain, leading to destabilization of the redox status of the cells. Genes related to glycogen metabolism (ECA4148-ECA4151) were downregulated in the mutant strain (Table S1). This is consistent with the fact that RpoS is a positive regulator of glycogen metabolism, and the Δ*rpoS* mutant of *P. carotovorum* was deficient in glycogen accumulation [21]. In turn, the metabolism of glycogen is known to be an important aspect of bacterial adaptation [90]; therefore, its reduction can decrease bacterial fitness under adverse conditions.

Among 476 transporter-related genes, 87 were DEGs in the mutant vs. WT under either growth or starvation conditions (or under both conditions), indicating that RpoS is likely to play a significant role in coordinating the transport of amino acids, carbohydrates, inorganic substances, and some other compounds (Table S1). The participation of RpoS in the regulation of the expression of genes encoding the transporters of amino acids, oligopeptides, sugars, and ions (including iron, magnesium, and sulfur) was previously registered in *E. coli* and *Salmonella enterica* [16,91].

Interestingly, none of the 27 genes upregulated in the mutant vs. WT under starvation (Mu_S/WT_S) were DEGs in the mutant vs. WT under growth conditions (Mu_G/WT_G), and none of the 21 genes upregulated in the mutant vs. WT under growth conditions (Mu_G/WT_G) were DEGs in the mutant vs. WT under starvation (Mu_S/WT_S) (Table S1). Similarly, only three genes were simultaneously downregulated in the mutant vs. WT under both assayed conditions, whereas 21 and 15 genes were downregulated in the mutant vs. WT solely under starvation or growth conditions, respectively (Table S1). This means that the regulatory effect of RpoS on transport processes totally differs depending on environmental conditions.

Importantly, among the 24 transporter-related genes downregulated in the mutant vs. WT under starvation (Mu_S/WT_S) (and thus positively regulated by RpoS), 21 (88%) were upregulated in the WT under starvation (WT_S/WT_G) (Table S1). In contrast, among the 27 genes upregulated in the mutant vs. WT under starvation (Mu_S/WT_S) (and thus negatively regulated by RpoS), only 6 (22%) were upregulated in the WT under starvation (WT_S/WT_G) (Table S1). This means that RpoS positively regulates those transport-related genes whose enhanced expression is associated with the starvation response.

#### 2.2.5. Verification of RNA-Seq Data

**Figure 4:**
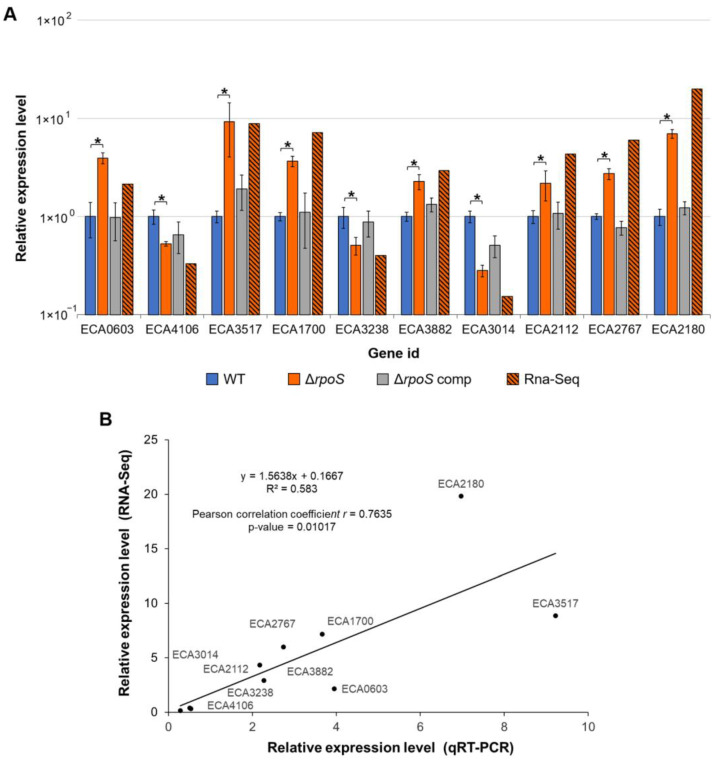
Gene expression levels in the wild-type *Pectobacterium atrosepticum* SCRI1043 (WT, blue columns), *P. atrosepticum* Δ*rpoS* mutant (Δ*rpoS*, orange columns), and complemented Δ*rpoS* mutant carrying the *rpoS* gene within the recombinant plasmid (Δ*rpoS* comp, gray columns); all strains were incubated under starvation for one day. (**A**) The expression levels of each gene in the WT are equated to one, and the expression levels in the Δ*rpoS* mutant and complemented mutant are presented relative to the WT. Gene expression levels were determined by quantitative polymerase chain reaction (qRT-PCR). The hatched orange-black columns show the gene expression levels determined by RNA-Seq in the Δ*rpoS* mutant relative to the WT under starvation conditions. Asterisks show the significance of the difference in the mean values of gene expression levels in Δ*rpoS* mutant and WT under starvation (two-sided *t*-test with Benjamini–Hochberg adjustment, FDR < 0.05). (**B**) Correlation between gene expression levels determined by qRT-PCR and RNA-Seq in the Δ*rpoS* mutant vs. WT under starvation.

To verify our RNA-Seq data, we selected 10 DEGs that were either up- or downregulated in the mutant compared to the WT under starvation conditions and determined the expression levels of these genes using qPCR. When analyzed by qPCR, all selected genes showed statistically significant differences in expression levels in the mutant vs. WT under starvation and displayed expression patterns similar to those obtained using RNA-Seq. RNA-Seq and qPCR data showed a high level of correlation (Pearson correlation coefficient *r* = 0.7635, *p*-value 0.01017) (Figure 4). In addition, we determined the expression levels of these genes in the complemented mutant carrying the *rpoS* gene within the recombinant plasmid and showed that complementation restored gene expression values in the mutant to the WT level (Figure 4).

**Figure 5:**
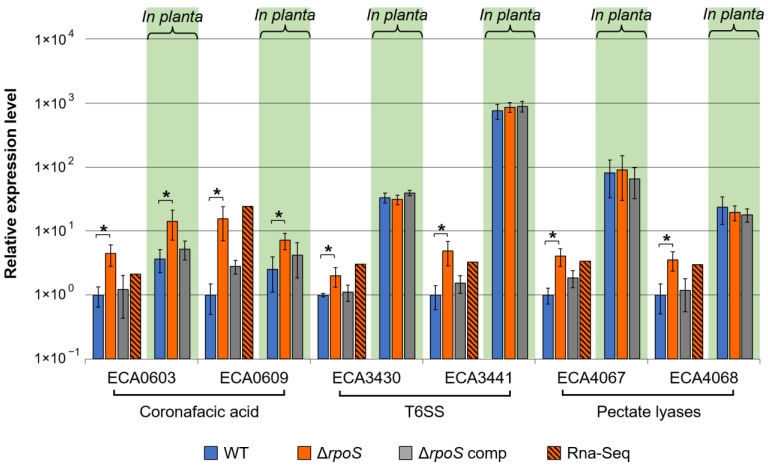
Gene expression levels in the wild-type *Pectobacterium atrosepticum* SCRI1043 (WT, blue columns), *P. atrosepticum* Δ*rpoS* mutant (Δ*rpoS*, orange columns), and complemented Δ*rpoS* mutant carrying the *rpoS* gene within the recombinant plasmid (Δ*rpoS* comp, gray columns); all strains were either grown in vitro until the early stationary phase (columns with no color background) or inoculated into tobacco plants and propagated within plants for two days until visible symptom expression (columns on green background, in planta). The expression levels of each gene in the WT grown in vitro are equated to one, and the expression levels in the Δ*rpoS* mutant and complemented mutant grown in vitro, as well as in all three strains propagated in planta, are presented relative to the WT grown in vitro. Gene expression levels were determined by quantitative polymerase chain reaction (qRT-PCR). The hatched orange-black columns show the gene expression levels determined by RNA-Seq in the Δ*rpoS* mutant relative to the WT under in vitro growing conditions. Asterisks show the significance of the difference in the mean values of gene expression levels in Δ*rpoS* mutant and WT under in vitro or in planta conditions (two-sided *t*-test with Benjamini–Hochberg adjustment, FDR < 0.05).

Although most of the DEGs in the mutant vs. WT were revealed under starvation conditions, many virulence-associated genes related to coronafacic acid biosynthesis and T6SS were upregulated in the mutant vs. WT under growth conditions (Mu_G/WT_G), as well as two genes encoding pectate lyases, the major *P. atrosepticum* virulence factors. Therefore, we also verified the expression levels of two coronafacic acid-related, two T6SS-related, and two pectate lyase-encoding genes (all were upregulated in the mutant vs. WT under growth conditions according to the RNA-Seq data) using qPCR in the WT, mutant, and complemented mutant under growth conditions (Figure 5). In addition, we questioned whether the negative RpoS-mediated regulation of the expression of these genes is also manifested when *P. atrosepticum* colonizes host plants, and therefore, we compared the expression levels of these six genes in three assayed strains under in planta conditions (in tobacco plants, two days post-inoculation, when disease symptoms were well-manifested) (Figure 5).

The data obtained by qPCR corresponded to the results of RNA-Seq: all six analyzed genes were upregulated in the mutant vs. WT under growth conditions (Figure 5). The complementation of the mutant with a plasmid carrying the *rpoS* gene restored the gene expression levels to those of the WT. All six genes were upregulated in the WT under in planta conditions (compared to in vitro growth conditions). Interestingly, genes related to the biosynthesis of coronafacic acid were upregulated in the mutant vs. WT not only under in vitro conditions but also under in planta conditions (Figure 5), indicating that the negative regulatory effect of RpoS on coronafacic acid production may be manifested during plant–pathogen interaction. Herewith, the transcript levels of T6SS-related and pectate lyase-encoding genes were similar in all three strains under in planta conditions (Figure 5), indicating that the negative regulatory effect of RpoS is manifested only in vitro but not during host colonization when the expression of these genes is strongly induced by many other regulatory systems, including those that perceive host plant metabolites [92,93].

Thus, our qPCR data on gene expression, including those that show the restoration of the expression level in the complementation mutant to the WT level, strongly confirm the obtained RNA-Seq results. The negative RpoS-mediated regulation observed for some virulence-related genes (T6SS-related and pectate lyase-encoding) is manifested only under in vitro growth conditions but did not occur under in planta conditions, where these genes are strongly upregulated compared to in vitro growth conditions. Herein, the expression of genes related to the synthesis of coronafacic acid, an important *P. atrosepticum* virulence factor that promotes host plant susceptibility and aggressive pathogen behavior [41,84,85], is subjected to an obvious negative regulation by RpoS, which is manifested under all three assayed conditions (growth in vitro, starvation, and in planta).

#### 2.2.6. Genes of the *P. atrosepticum* RpoS Regulon

**Figure 6:**
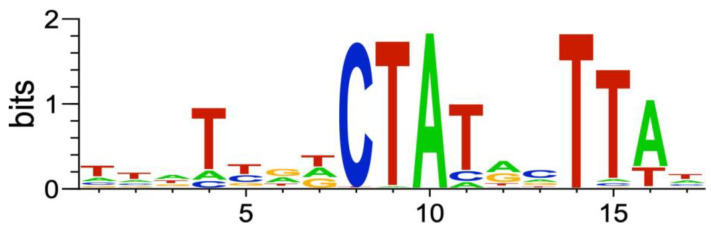
Sequence logo of the predicted RpoS promoter motif in *Pectobacterium atrosepticum* SCRI1043.

To predict genes/operons that are directly regulated by RpoS in *P. atrosepticum* based on the obtained RNA-Seq data, we searched for conservative motifs within the promoter regions of the *P. atrosepticum* RpoS-regulated genes. The found motif (Figure 6) resembled the *P. atrosepticum* RpoS promoter motif inferred in our previous study based on indirect evidence [94].

Promoters matching the DEG-based motif (Figure 6) were found upstream of 65 operons comprising 122 genes (Table S2). These 122 genes are further referred to as genes of the *P. atrosepticum* RpoS regulon. Operons with an RpoS-dependent promoter contained one to seven genes. Genes of the *P. atrosepticum* RpoS regulon belonged to the supercategories “General metabolism” (36), “Unknown” (35), “Stress” (20), “Transporters” (14), “Other” (13), “Envelope” (genes related to lipopolysaccharide biosynthesis) (11), “Regulators” (5, all encoding TFs), “Virulence” (4), and “Motility” (1); herewith, note that eight genes of the *P. atrosepticum* RpoS regulon were simultaneously attributed to 2–4 supercategories (Table S2).

Genes of the *P. atrosepticum* RpoS regulon from the supercategory “General metabolism” were mostly related to amino sugar and nucleotide sugar metabolism, citrate cycle, aerobic respiration, pyruvate metabolism, and protein metabolism (Table S2). Within the “Stress” supercategory, the *P. atrosepticum* RpoS regulon included genes from the categories “Antibiotics/Multidrug resistance” (eight), “Stress proteins” (six), “Antioxidant systems” (four, including two genes encoding superoxide dismutases SodA (ECA0092) and SodC (ECA2211)), “Degradation of xenobiotics” (one), and “Toxin-antitoxin system” (one) (Table S2). Six genes from the category “Stress proteins” attributed to the *P. atrosepticum* RpoS regulon encode the universal stress protein UspA (ECA0050), envelope stress response membrane protein PspB (ECA1984), stress-induced protein YchH (ECA2185), stress response protein ElaB (ECA3015), entericidin A/B family lipoprotein EcnB (ECA3975), and periplasmic chaperone OsmY (ECA0469) (Table S2).

Overall, genes with presumed direct transcriptional control via RpoS (*P. atrosepticum* RpoS regulon) represented a minor portion of the total DEGs revealed for the mutant vs. WT, indicating that most of these DEGs were regulated by RpoS in an indirect manner. Such indirect regulation can be achieved via TFs, whose gene expression is controlled by RpoS. Among the four TF-encoding genes attributed to the *P. atrosepticum* RpoS regulon, two, HyfR (ECA1236) and PspC (ECA1985), were positively regulated by RpoS in our experiments under starvation conditions (downregulated in Mu_S/WT_S) (Table S2). Therefore, these TFs are prime candidates for consideration from the viewpoint of mediating RpoS effects under starvation.

Thus, the predicted *P. atrosepticum* RpoS regulon is most represented by genes related to general metabolism. Herewith, stress-related genes, including those encoding antioxidant proteins and stress proteins, are also present in the predicted *P. atrosepticum* RpoS regulon. Many genes encoding transporters appeared among the genes of the predicted *P. atrosepticum* RpoS regulon.

The RpoS promoter motif inferred in our study for *P. atrosepticum* (Figure 6) was similar to the well-defined *E. coli* RpoS promoter motif [95]. Since only the −10 promoter regions are conserved, and the amino acid residues responsible for the recognition of the DNA bases within this region are identical between the RpoS of *E. coli* and *P. atrosepticum*, we analyzed the similarity/dissimilarity of the predicted *P. atrosepticum* RpoS regulon with a well-characterized RpoS regulon of *E. coli* [96]. For 80 of the 122 genes in the predicted *P. atrosepticum* RpoS regulon, orthologous genes were found in *E. coli* (Table S2). Of these 80 *E. coli* genes, only 13 belonged to the RpoS regulon of *E. coli*, according to RegulonDB [96].

Thus, only 10.7% (13 of 122) of the *P. atrosepticum* RpoS regulon genes were shared among the RpoS regulons of *P. atrosepticum* and *E. coli* (Table S2). These shared genes were exclusively represented by genes from two gene supercategories: “General metabolism” (including a gene operon ECA3969-ECA3972 encoding subunits of the citrate cycle enzyme fumarate reductase) and “Stress” (Table 6 and Table S2). The stress-related gene encoding copper-zinc superoxide dismutase SodC (ECA2211) has the most evident features of the RpoS-regulated gene: it was found in a “core” RpoS regulon of *P. atrosepticum* and *E. coli* and was positively regulated by RpoS in *P. atrosepticum* under both assayed conditions (downregulated in Mu_S/WT_S and Mu_G/WT_G) (Table 6 and Table S2).

## 3. Materials and Methods

### 3.1. Bacterial Strains, Media, and Culture Conditions

*Pectobacterium atrosepticum* SCRI1043, its Δ*rpoS* mutant, and complemented Δ*rpoS* mutant were routinely grown in lysogeny broth (LB) medium on a rotary shaker (180 rpm) at 28 °C. The Δ*rpoS* mutant strain was grown in the presence of kanamycin (20 μg mL^−1^), and the complemented Δ*rpoS* mutant was grown in the presence of kanamycin (20 μg mL^−1^) and ampicillin (200 μg mL^−1^). The colony-forming unit (CFU) titer was determined by plating serial 10-fold dilutions of the cultures onto 1.5% LB agar. For the starvation assay, the modified medium D5 without a carbon source (0.1 mM Na-K phosphate buffer (pH 7.5), 1.0 g L^−1^ NH_4_Cl, 0.3 g L^−1^ MgSO_4_·7H_2_O) was used. Bacterial cells of wild-type *P. atrosepticum* (WT), Δ*rpoS* mutant, or complemented Δ*rpoS* mutant were harvested (8000× *g*, 10 °C, 10 min) at the early stationary growth phase (1–2 × 10^9^ CFU mL^−1^), then washed twice in a carbon-free D5 medium. The cells were then resuspended in a carbon-free D5 medium up to a density of 6–8 × 10^8^ CFU mL^−1^. The resultant starving cultures were incubated in glass vials without aeration at 28 °C. For gene expression analyses (by RNA-Seq and qRT-PCR), as well as stress tolerance and virulence assays, bacteria of all three strains were either grown in LB medium without antibiotics until the early stationary phase (growth conditions) or incubated without antibiotics in a carbon-deficient D5 medium for one day (starvation conditions).

### 3.2. Construction of rpoS Deletion Mutant

The *rpoS* deletion mutant (Δ*rpoS*) was constructed using a previously described method [97]. The target gene *rpoS* (ECA3530 locus), together with the adjacent regions (approximately 1000 bp up- and downstream of *rpoS* ORF), was amplified by PCR with the primers up*rpoS*F and dn*rpoS*R (Table S3) using Q5 high-fidelity DNA polymerase (NEB, Ipswich, MA, USA). The amplified PCR fragment was cloned into the bacterial cloning vector system pGEM-T Easy (Promega, Madison, WI, USA). The obtained plasmid (pGEM::*rpoS*) was introduced into *E. coli* NovaBlue by chemical transformation. Transformants carrying the recombinant plasmid were screened for ampicillin resistance and further verified by PCR using plasmid-specific primers for the T7 and SP6 polymerase promoters (Table S3), which flank the multiple cloning regions of pGEM-T Easy.

To replace the 589-bp fragment of the *rpoS* ORF with the Km^R^ cassette, a part of the pGEM::*rpoS* plasmid (excluding 589 bp regions of the *rpoS* ORF) was amplified with the primers dn*rpoS*KmF and up*rpoS*KmR (Table S3), whose 5′-ends were complementary to the end regions of the Km^R^ cassette. The amplified PCR fragment was treated with restriction endonuclease DpnI (NEB, Ipswich, MA, USA) to remove the original methylated plasmid and then purified using a DNA cleanup kit (NEB, Ipswich, MA, USA). The Km^R^ cassette was amplified from the pKD4 plasmid using the primers Km*rpoS*F and Km*rpoS*R (Table S3), whose 5′-ends were complementary to *P. atrosepticum* DNA regions adjacent to the *rpoS* ORF. Two obtained PCR fragments (corresponding to the pGEM plasmid with ~1000 bp regions up- and downstream of the *rpoS* ORF and to the KmR cassette) were joined by the circular polymerase extension cloning method [98]. The obtained plasmid (pGEM::Δ*rpoS*; Km^R^) was introduced into *E. coli* NovaBlue by chemical transformation. The mutant locus was confirmed by DNA sequencing.

The mutant locus (containing the Km^R^ cassette and ~1000 bp regions up- and downstream of the *rpoS* ORF) was amplified with the primers up*rpoS*F and dn*rpoS*R (Table S3) and ligated (T4 ligase, NEB, Ipswich, MA, USA) into the SmaI-digested (NEB, Ipswich, MA, USA) suicide vector pKNG101 to generate the recombinant plasmid containing the allelic exchange cassette for the target locus. The obtained plasmid (pKNG101::Δ*rpoS*; Km^R^) was introduced into *E. coli* CC118 by electroporation. Transfer of the pKNG101::Δ*rpoS* plasmid from *E. coli* CC118 into *P. atrosepticum* was achieved by triparental mating using *E. coli* HH26 as a helper strain. Clones in which the pKNG101::Δ*rpoS* plasmid was integrated into the chromosome by a single crossover event were selected by streptomycin and kanamycin resistance. Clones with the second crossover event replacing the target locus with the mutant locus and with the elimination of the donor plasmid were selected on M9 agar medium containing 10% sucrose. The clones were then tested for streptomycin sensitivity. Streptomycin-sensitive clones were analyzed by PCR using the primers Check*rpoS*F and Check*rpoS*R (Table S3) to identify Δ*rpoS* mutants.

### 3.3. Construction of the ΔrpoS Complemented Mutant Strain Carrying the rpoS Gene on the Plasmid

To construct the complementing plasmid, the 1326-bp region containing the *rpoS* gene was amplified by PCR with primers comp*rpoS*F and comp*rpoS*R (Table S3) using Q5 high-fidelity DNA polymerase (NEB, Ipswich, MA, USA). The amplified fragment was cloned into the bacterial cloning vector pGEM-T Easy (Promega, Madison, WI, USA). The obtained plasmid (pGEM::*rpoS*;Amp^R^) was introduced into *E. coli* NovaBlue by chemical transformation. Transformants carrying the recombinant plasmid were screened for ampicillin resistance and further verified by PCR using plasmid-specific primers for the T7 and SP6 polymerase promoters. The correctness of the assembly was confirmed by DNA sequencing.

The pGEM::*rpoS* plasmid was introduced into the *P. atrosepticum* Δ*rpoS* mutant by electroporation. Clones with ampicillin and kanamycin resistance were analyzed by PCR using the primers Km*rpoS*F/Km*rpoS*R and *rpoS*F/*rpoS*R (Table S3) to confirm the presence of the complementing plasmid.

### 3.4. Stress Tolerance Assay

To compare the stress resistance of the WT, Δ*rpoS* mutant, and complemented Δ*rpoS* mutant, bacteria were cultured in LB medium until the early stationary phase or incubated starting with an initial population density of 6–8 × 10^8^ CFU mL^−1^ in a carbon-free D5 medium for one day. Bacterial cells were harvested (8000× *g*, 10 °C, 10 min), and cell densities of different *P. atrosepticum* cultures were adjusted to 10^8^ CFU mL^−1^ by diluting in D5 medium. For the heat challenge, 100 µL aliquots of cell suspensions were exposed to 48 °C for 5 min. Fifteen min after heat shock, suspensions were plated onto 1.5% LB agar as serial 10-fold dilutions. For the oxidative or antibiotic challenge, bacteria were incubated in the presence of H_2_O_2_ (2.5 mM) or rifampin (40 μg mL^−1^). These concentrations of H_2_O_2_ and rifampin, as well as the temperature, were previously selected for the optimal differentiation of *P. atrosepticum* cultures according to cell resistance [31]. After 24 h of incubation, the suspensions were plated onto LB agar plates and incubated at 28 °C for 2 days before the CFUs were counted.

### 3.5. Plant Cultivation and Infection

*Nicotiana tabacum* cv. Petit Havana SR1 plants were grown axenically in test tubes in a growth chamber with a 16 h light/8 h dark cycle photoperiod. Seeds were surface-sterilized using diluted bleach (0.8% of active chlorine) and 1% sodium dodecyl sulfate for 30 min, washed seven times with sterile distilled water, and then transferred to Murashige and Skoog medium (MS) in Petri dishes. Ten-day-old seedlings were transferred to individual flasks containing MS.

Plants (five weeks after planting) were infected with WT, Δ*rpoS* mutant, or complemented Δ*rpoS* mutant. For plant inoculation, bacteria were grown until the early stationary phase (1–2 × 10^9^ CFU mL^−1^) or incubated starting with an initial population density of 6–8 × 10^8^ CFU mL^−1^ in carbon-free D5 medium for one day (starving cultures), then washed with sterile 10 mM MgSO_4_ and resuspended in the same solution up to a density of ~2 × 10^7^ CFU mL^−1^. Sterile 10 mM MgSO_4_ or bacterial suspensions containing ~2 × 10^5^ cells were placed as 10 μL drops on leaves using sterile pipette tips.

For plant priming, plants were sprayed with 200 µL of 0.2 mM salicylic acid (SA). The SA solution was sterilized by filtration through 0.2 µm pore sterile filters (Corning, Berlin, Germany). SA treatment was performed one day before the infection. Disease incidents were scored 1, 2, 4, and 6 days after infection. The results were obtained from three independent experiments; in each experiment, 30 plants were assessed for each experimental variant.

### 3.6. RNA Extraction

Total RNA was extracted from bacterial cells grown or incubated in vitro (for RNA-Seq and qPCR analyses) or from plants infected by the WT, Δ*rpoS* mutant, or complemented Δ*rpoS* mutant two days after plant inoculation when disease symptoms were well-manifested (for qRT-PCR analysis). Plant material ground in liquid nitrogen in mortars or bacterial cell pellets was resuspended in 1 mL of ExtractRNA Reagent (Evrogen, Moscow, Russia), and the subsequent procedures were performed according to the manufacturer’s instructions. RNA samples were treated with DNAse I using the DNA-free kit (Thermo Fisher Scientific, Waltham, MA, USA). RNA quantity and quality were analyzed using a Qubit fluorimeter (Life Technologies, Carlsbad, CA, USA) and a Qsep100 DNA Analyzer (Bioptic, New Taipei City, Taiwan), respectively.

### 3.7. cDNA Library Preparation and Sequencing

For RNA-Seq, total RNA (2 μg) was processed using a RiboMinus Bacteria 2.0 Transcriptome Isolation Kit (Thermo Fisher Scientific, Waltham, MA, USA) and then a NEBNext^®^ Ultra™ II Directional RNA Library Prep Kit for Illumina (NEB, Ipswich, MA, USA) according to the manufacturer’s instructions. The quality and quantity of the cDNA libraries during processing before sequencing were monitored using an Agilent 2100 Bioanalyzer (Agilent, Santa Clara, CA, USA) and a CFX96 Touch Real-Time PCR Detection System (Bio-Rad, Hercules, CA, USA). Libraries were sequenced in three biological replicates. Sequencing was performed on an Illumina HiSeq 2500 platform (Illumina, San Diego, CA, USA) at the Joint KFU–Riken Laboratory, Kazan Federal University (Kazan, Russia).

### 3.8. qRT-PCR Analysis

One microgram of DNAse-treated RNA was used for cDNA synthesis using RevertAid reverse transcriptase (Thermo Fisher Scientific, Waltham, MA, USA) according to the manufacturer’s instructions. Two microliters of 5-fold-diluted cDNA preparation were used for qPCR. qPCR was performed using the EVAGreen-containing master mix (Syntol, Moscow, Russia) according to the manufacturer’s instructions.

Primers for target and reference genes (Table S3) were designed using Vector-NTI Version 9 software (Invitrogen, Carlsbad, CA, USA) and synthesized by Evrogen (Moscow, Russia). Genes encoding RNA polymerase sigma factor RpoD (ECA0680 locus), signal recognition particle protein Ffh (ECA3360 locus), and protein RecA (ECA3369 locus), the transcript level of which was confirmed by geNorm software (http://genorm.cmgg.be/, accessed on 7 September 2023) to be stable under the experimental conditions, were used for normalization of the target gene expression. Relative expression levels were determined as the ratios between the quantities of cDNA corresponding to the target genes and values of the normalization factor, which was calculated for each sample using geNorm software v. 3.5 based on the transcript levels of reference genes. The presented data were obtained by analyzing 5–8 biological replicates.

### 3.9. Transcriptome Analysis

Raw reads obtained in our study are available at the NCBI BioProject under the accession number PRJNA1041790. The quality of the reads was assessed using FastQC (http://www.bioinformatics.babraham.ac.uk/projects/fastqc/, accessed on 4 March 2023). Illumina adapters were removed using the bbduk tool (https://jgi.doe.gov/data-and-tools/software-tools/bbtools/bb-tools-user-guide/bbduk-guide/, accessed on 4 March 2023). Low-quality reads (q-score < 30) and reads corresponding to rRNAs were filtered out using Trimmomatic and SortMeRNA, respectively [99,100]. Pseudo-alignment on a reference sequence (*P. atrosepticum* SCRI1043 coding sequence (CDS) NCBI Assembly GCF_000011605.1) and quantification of filtered reads were carried out using kallisto [101]. Differentially expressed genes (DEGs) were identified using the edgeR package v. 3.40.2 [102]. Genes with TMM-normalized read counts per million (CPM) values less than one under all experimental conditions were considered to be non-expressed in our study. Genes with |log_2_ FC| > 1 and a false discovery rate (FDR) < 0.05 were considered to be DEGs. Gene functional classification performed in our previous study [84] was used in our present study with some modifications related to the updated annotations of *P. atrosepticum* SCRI1043 genes; the functional classification of genes encoding transcription factors was modified according to the analysis performed by the TF classifier tool of BacRegDB (http://bacregdb.bsu.by/tools?toolName=classify, accessed on 17 August 2023).

### 3.10. P. atrosepticum RpoS Regulon Inference

MEME [103] v. 5.5.4 was used for inference of conserved motifs within promoter regions of *P. atrosepticum* genes positively regulated by RpoS. The resulting motif was used to search for RpoS-dependent promoters in the *P. atrosepticum* genome with SigmoID v. 2.0b2 [94]. SigmoID was also used for calculating and visualizing sequence logos. RNA-seq coverage plots were used to verify the positions of the putative promoters. Coverage plots were generated with samtools v. 1.18 [104] from RNA-Seq data mapped onto *P. atrosepticum* genome with BWA v. 0.7.17 [105] and visualized with SigmoID. The number of genes within each operon located downstream of the RpoS promoter was determined with SigmoID based on promoter and terminator positions, a web-server for accurate prediction of prokaryotic operons, “Operon Finder” [106], and RNA-seq coverage profiles.

The comparison of the RpoS regulon of *P. atrosepticum* predicted in our study with the previously determined RpoS regulon of *E. coli* was performed using InParanoid-DIAMOND (https://bitbucket.org/sonnhammergroup/inparanoid/src/master/, accessed on 10 September 2023) [107] and RegulonDB (https://regulondb.ccg.unam.mx/sigmulon/RDBECOLISFC00007, accessed on 10 September 2023). First, *E. coli* genes orthologous to genes of the *P. atrosepticum* RpoS regulon were searched using InParanoid-DIAMOND based on the analysis of the reference proteomes of *P. atrosepticum* (https://www.ncbi.nlm.nih.gov/datasets/genome/?taxon=218491, file RefSeq, accessed on 10 September 2023) and *E. coli* (https://www.uniprot.org/proteomes/UP000000625, accessed on 10 September 2023). Then, among *E. coli* genes orthologous to genes of the *P. atrosepticum* RpoS regulon, the genes that belong to the RpoS regulon of *E. coli* were selected using RegulonDB; herewith, only those *E. coli* genes for which the evidence type for the presence of the RpoS-dependent promoter corresponded to “Strong Evidence” were considered.

### 3.11. Motility Assays

To test the motility of bacterial cells (*P. atrosepticum*, its Δ*rpoS* mutant, and complemented Δ*rpoS* mutant), semi-solid LB medium (0.4% of agar) was inoculated with 3 µL of bacterial suspensions containing ~10^6^ cells that were placed into the center of a Petri dish. Seeded Petri dishes were incubated at 28 °C, and the diameters of the macrocolonies were measured four days after inoculation. The experiment was performed in five biological replicates.

## 4. Conclusions

Our study shows that RpoS in *P. atrosepticum* contributes to long-term survival under starvation and the formation of cross-protection. Additionally, RpoS provides *P. atrosepticum* with the ability to cause disease in primed plants with enhanced immune status. However, even without a functional *rpoS* gene, *P. atrosepticum* cells retain the ability to cause disease in non-primed plants, adapt to starvation, and form at least some level of cross-protection following primary stress. This shows that there is an RpoS-independent “side” of *P. atrosepticum* adaptation. In particular, the maintenance of the virulence potential during stress exposure is implemented in an RpoS-independent manner since the starved cells of the WT and the *rpoS* mutant show similar virulence levels despite their different levels of cross-protection.

Our study, for the first time in phytopathogenic bacteria, considered the regulatory effect of RpoS at the whole-transcriptome level. Although RpoS is often considered to be the regulator that provides enhanced expression of stress-related genes, in our study, more stress-related genes were negatively regulated by RpoS than were positively regulated. This casts doubt that the major regulatory role of RpoS in *P. atrosepticum* is the activation of stress protein production at the expense of different metabolic pathways to increase cell stress resistance. However, some stress-related genes were indeed positively controlled by RpoS, and among these genes, *sodC*, which encodes copper-zinc superoxide dismutase, has the most evident features of the RpoS-regulated gene.

Based on the obtained RNA-Seq data, we inferred the RpoS promoter motif and predicted 65 operons involving 122 genes, presumably directly regulated by RpoS in *P. atrosepticum* and thus comprising the *P. atrosepticum* RpoS regulon. Genes of the predicted *P. atrosepticum* RpoS regulon comprise only a minor portion of the total DEGs revealed in the mutant vs. WT comparison, indicating that most of these DEGs are regulated by RpoS indirectly. We found two TF-encoding genes that belonged to the predicted *P. atrosepticum* RpoS regulon and were downregulated in the *rpoS* mutant vs. WT (thus positively regulated by RpoS) in our experiments. Therefore, these TFs are prime candidates to consider from the perspective of their potential role in mediating the effect of RpoS and acting downstream of RpoS to contribute to adaptation and the formation of cross-protection. Additionally, we found several TF-encoding genes without detectable RpoS-dependent promoters that were positively regulated by RpoS under starvation and upregulated during starvation in the WT. These TFs, although less likely to be directly controlled by RpoS, also deserve further investigation as potential regulators of the RpoS-dependent starvation response.

RpoS negatively affects the expression of many virulence-associated genes in *P. atrosepticum* under growth conditions. In addition to the negative effect of RpoS on the expression of pectate lyase genes (also noted for *Pectobacterium* species in previous studies), RpoS also negatively affected the expression levels of many genes related to motility (resulting in a hypermotile phenotype of the *rpoS* mutant) and the T6SS. The most pronounced negative effect of RpoS on virulence-associated *P. atrosepticum* genes was observed for coronafacic acid biosynthesis-related genes; this effect (in contrast to the effects on other virulence-associated genes) was manifested under all studied conditions (growth, starvation, and in planta). However, such a negative effect of RpoS on the expression of virulence-related genes did not result in the hypervirulent phenotype of the *rpoS* mutant. The lack of enhanced virulence in the *rpoS* mutant can presumably be explained by the reduced stress resistance of the mutant, which neutralizes the increased expression of virulence-associated genes. The attenuated virulence of the *rpoS* mutant toward primed plants supports this assumption.

Many genes whose expression was regulated by RpoS in *P. atrosepticum* are related to general metabolism, predominantly to the metabolism of proteins, amino acids, and glycogen. Genes related to general metabolism were also the most represented in the predicted *P. atrosepticum* RpoS regulon. Interestingly, 9 of the 13 genes shared between the predicted *P. atrosepticum* RpoS regulon and a well-defined RpoS regulon of *E. coli* are related to general metabolism. A large portion of transporter-encoding genes were regulated in the *rpoS* mutant compared to the WT. Herewith, almost all transporter-encoding genes were regulated under either growth or starvation conditions but not under both conditions simultaneously. A possible global effect of RpoS on the transport of different compounds and ions due to the observed condition-specific regulation of the expression of many transporter-encoding genes can cause dramatic changes in physiological processes, including those originating from alterations in membrane permeability. This, in turn, can lead to downstream regulation of the expression of many genes and the determination of the RpoS-dependent phenotype. Therefore, without denying the role of RpoS in regulating the expression of some stress-related genes important for adaptation, due to the revealed effects of RpoS on general metabolism- and transport-process-related genes, we incline to the previously stated hypothesis that RpoS is the second (after RpoD) vegetative sigma factor whose primary role consists of the regulation of primary metabolism [74,108,109].

Our results agree with the current opinion that RpoS regulons are highly variable in different bacterial species [11]. In our study, only 10.7% of genes were shared between the predicted *P. atrosepticum* RpoS regulon and the RpoS regulon of *E. coli*. Further accumulation of data on the RpoS regulons of other phytopathogenic bacteria would enable the search for common and distinctive features between them. RpoS-mediated regulation in *P. atrosepticum* also deserves further consideration using various test systems (including those involving the application of different stressors) to gain deeper insight into the role of RpoS in bacterial physiology and come closer to understanding the mechanisms of the RpoS-independent “side” of bacterial adaptation.

## Figures and Tables

**Table 1:** The number of differentially expressed genes (DEGs) (up- or downregulated) in wild-type *Pectobacterium atrosepticum* SCRI1043 (WT) under starvation versus WT during growth (WT_S/WT_G); Δ*rpoS* mutant of *P. atrosepticum* SCRI1043 under starvation compared to Δ*rpoS* mutant during growth (Mu_S/Mu_G); Δ*rpoS* mutant under starvation compared to WT under starvation (Mu_S/WT_S); Δ*rpoS* mutant during growth compared to WT during growth (Mu_G/WT_G).

Variant of Comparison	Total DEGs	Upregulated DEGs	Downregulated DEGs
WT_S/WT_G	2222	1075	1147
Mu_S/Mu_G	1934	968	966
Mu_S/WT_S	615	389	226
Mu_G/WT_G	359	200	159

**Table 2:** Expression levels of genes from the category “Stress proteins” that were upregulated in the Δ*rpoS* mutant of *Pectobacterium atrosepticum* SCRI1043 compared to wild-type *P. atrosepticum* SCRI1043 (WT) during starvation. Red and blue backgrounds mark the up- and downregulated genes, respectively, within different variants of comparison: WT under starvation versus WT during growth (WT_S/WT_G); Δ*rpoS* mutant of *P. atrosepticum* SCRI1043 under starvation compared to Δ*rpoS* mutant during growth (Mu_S/Mu_G); Δ*rpoS* mutant under starvation compared to WT under starvation (Mu_S/WT_S); and Δ*rpoS* mutant during growth compared to WT during growth (Mu_G/WT_G). nD—non-differentially expressed gene.

			Expression Level, log_2_FC			
Locus Tag	Gene	Annotation	WT_S/ WT_G	Mu_S/ Mu_G	Mu_S/ WT_S	Mu_G/ WT_G
ECA2767	*dps*	DNA starvation/stationary phase protection protein Dps	nD	3.31	2.58	nD
ECA0443		Txe/YoeB family addiction module toxin	1.29	1.71	1.11	nD
ECA0900	*tehB*	SAM-dependent methyltransferase TehB	nD	2.69	1.83	nD
ECA3053	*cvpA*	colicin V production protein	nD	1.55	2.11	nD
ECA1580		cold-shock domain-containing protein	−1.03	nD	1.1	nD
ECA3527	*rcsF*	Rcs stress response system protein RcsF	−3.63	−1.82	1.48	nD
ECA2659	*cspD*	cold-shock-like protein CspD	nD	nD	1.54	1.46
ECA4402	*ibpB*	heat-shock chaperone IbpB	nD	2.89	2.45	−1.07
ECA4403	*ibpA*	heat-shock chaperone IbpA	nD	3.34	2.75	nD

**Table 3:** Expression levels of genes from the supercategory “Stress” that were similarly regulated under growth and starvation conditions in the Δ*rpoS* mutant of *Pectobacterium atrosepticum* SCRI1043 compared to wild-type *P. atrosepticum* SCRI1043 (WT). Red and blue backgrounds mark the up- and downregulated genes, respectively, within different variants of comparison: WT under starvation versus WT during growth (WT_S/WT_G); Δ*rpoS* mutant of *P. atrosepticum* SCRI1043 under starvation compared to mutant during growth (Mu_S/Mu_G); mutant under starvation compared to WT under starvation (Mu_S/WT_S); mutant during growth compared to WT during growth (Mu_G/WT_G). nD—non-differentially expressed gene.

			Expression Level, log_2_FC			
Locus Tag	Gene	Annotation	WT_S/ WT_G	Mu_S/ Mu_G	Mu_S/ WT_S	Mu_G/ WT_G
ECA3410		type I toxin–antitoxin system SymE family toxin	1.09	nD	−6.39	−6.72
ECA2211	*sodC*	copper-zinc superoxide dismutase	nD	nD	−2.19	−2.16
ECA0049	*uspB*	universal stress protein UspB	nD	nD	−1.66	−1.62
ECA3409		type I toxin–antitoxin system SymE family toxin	1.03	1.06	1.01	1.08
ECA2659	*cspD*	cold-shock-like protein CspD	nD	nD	1.54	1.46

**Table 4:** Expression levels of transcription factor (TF)-encoding genes that were both downregulated in the Δ*rpoS* mutant of *Pectobacterium atrosepticum* SCRI1043 compared to wild-type *P. atrosepticum* SCRI1043 (WT) under starvation (indicating that RpoS positively regulates their expression) and upregulated during starvation of the WT compared to the growth of the WT (indicating that these TF presumably participate in adaptation). Red and blue backgrounds mark the up- and downregulated genes, respectively, within different variants of comparison: WT under starvation versus WT during growth (WT_S/WT_G); Δ*rpoS* mutant of *P. atrosepticum* SCRI1043 under starvation compared to Δ*rpoS* mutant during growth (Mu_S/Mu_G); Δ*rpoS* mutant under starvation compared to WT under starvation (Mu_S/WT_S); Δ*rpoS* mutant during growth compared to WT during growth (Mu_G/WT_G). nD—non-differentially expressed gene.

			Expression Level, log_2_FC			
Locus Tag	Gene	Annotation	WT_S/ WT_G	Mu_S/ Mu_G	Mu_S/ WT_S	Mu_G/ WT_G
ECA2724	*rscR*	LysR-family transcriptional regulator	1.28	nD	−1.95	nD
ECA0739	*budR*	LysR-family transcriptional regulator	1.33	nD	−1.03	nD
ECA1806		helix-turn-helix domain-containing protein	1.42	nD	−1.24	nD
ECA0643	*exuR*	exu regulon transcriptional regulator	1.42	nD	−1.21	nD
ECA1985	*pspC*	phage shock protein C	1.47	nD	−1.16	nD
ECA0204	*rfaH*	transcription/translation regulatory transformer protein RfaH	1.58	nD	−1.23	nD
ECA0582		type II toxin–antitoxin system PemK/MazF family toxin	1.61	nD	−1.04	nD
ECA4389		PRD domain-containing protein	1.63	nD	−1.15	nD
ECA1849		GntR-family transcriptional regulator	1.64	nD	−1.00	nD
ECA0008	*qseB*	two-component system response regulator	1.66	nD	−1.40	nD
ECA1194	*cueR*	copper efflux regulator	1.70	nD	−1.64	nD
ECA3469		XRE family transcriptional regulator	2.06	nD	−1.08	nD
ECA4123	*rexZ*	regulator of exoenzymes	4.36	3.72	−1.02	nD
ECA0673		XRE family transcriptional regulator	4.43	3.30	−1.02	nD
ECA3238	*iscR*	Fe-S cluster assembly transcriptional regulator IscR	4.51	3.80	−1.32	nD
ECA0064		type II toxin–antitoxin system Phd/YefM family antitoxin	4.56	2.84	−1.75	nD
ECA4381	*lgoR*	L-galactonate utilization transcriptional regulator	5.47	3.68	−1.90	nD

**Table 5:** Expression levels of transcription factor (TF)-encoding genes that were both upregulated in the Δ*rpoS* mutant of *Pectobacterium atrosepticum* SCRI1043 compared to wild-type *P. atrosepticum* SCRI1043 (WT) under starvation and upregulated during starvation of the WT compared to the growth of the WT. Red backgrounds mark the up- and downregulated genes within different variants of comparison: WT under starvation versus WT during growth (WT_S/WT_G); Δ*rpoS* mutant of *P. atrosepticum* SCRI1043 under starvation compared to Δ*rpoS* mutant during growth (Mu_S/Mu_G); Δ*rpoS* mutant under starvation compared to WT under starvation (Mu_S/WT_S); Δ*rpoS* mutant during growth compared to WT during growth (Mu_G/WT_G). nD—non-differentially expressed gene.

			Expression Level, log_2_FC			
Locus Tag	Gene	Annotation	WT_S/ WT_G	Mu_S/ Mu_G	Mu_S/ WT_S	Mu_G/ WT_G
ECA4154		putative transcriptional regulator	1.03	2.68	1.60	nD
ECA3959		ArsR-family transcriptional regulator	1.07	1.86	1.43	nD
ECA0180	*metR*	transcriptional activator protein	1.54	4.43	3.12	nD
ECA2069		TetR-family transcriptional regulator	1.95	2.60	1.25	nD
ECA2295		AraC-family transcriptional regulator	1.95	3.52	1.68	nD
ECA0903	*norR*	nitric oxide reductase sigma-54-dependent transcriptional regulator	2.22	3.09	1.18	nD
ECA2910		MarR family transcriptional regulator	3.49	5.73	2.11	nD
ECA2873		putative transcriptional regulator	3.82	6.42	2.70	nD

**Table 6:** Genes shared in the predicted *P. atrosepticum* RpoS regulon (this study) and the RpoS regulon of *E. coli* (RegulonDB) [96].

Gene	Locus Tag		Product	Functional Supercategory
	*P. atrosepticum*	*E. coli*		
*osmY*	ECA0469	b4376	periplasmic chaperone	Stress
*alkB*	ECA0909	b2212	DNA oxidative demethylase	General metabolism
*galE*	ECA1389	b0759	UDP-glucose 4-epimerase	General metabolism
*galM*	ECA1388	b0756	galactose-1-epimerase	General metabolism
*pykF*	ECA1867	b1676	pyruvate kinase	General metabolism
*sodC*	ECA2211	b1646	superoxide dismutase (Cu-Zn)	Stress
*adhE*	ECA2326	b1241	fused acetaldehyde-CoA dehydrogenase and iron-dependent alcohol dehydrogenasealdehyde/alcohol dehydrogenase	General metabolism, stress
*elaB*	ECA3015	b2266	tail-anchored inner membrane protein	Stress
*frdA*	ECA3969	b4154	fumarate reductase flavoprotein subunit	General metabolism
*frdB*	ECA3970	b4153	fumarate reductase iron-sulfur protein	General metabolism
*frdC*	ECA3971	b4152	fumarate reductase membrane protein FrdC	General metabolism
*frdD*	ECA3972	b4151	fumarate reductase membrane protein FrdD	General metabolism
*ecnB*	ECA3975	b4411	bacteriolytic entericidin B lipoprotein	Stress

## Data Availability

Raw reads obtained in our study are available at the NCBI BioProject under the accession number PRJNA1041790.

## Supplementary Materials

The following supporting information can be downloaded at https://www.mdpi.com/article/10.3390/ijms242417348/s1. References [97,110,111,112,113] are cited in the supplementary materials.
