# Supplementary material for: RpoS-Regulated Genes and Phenotypes in the Phytopathogenic Bacterium Pectobacterium atrosepticum
Source: Int J Mol Sci. 2023 Dec 11;24(24):17348. doi: 10.3390/ijms242417348 (PMC10743746; doi:10.3390/ijms242417348)
Supplement: Supplementary file 1 [file ijms-24-17348-s001.zip › Table S3.pdf]

Table S3. Bacterial strains, plasmids and primers used in this study.

**Name**	**Description**	**Origin**
	**Strain**	
Pectobacterium atrosepticum SCRI1043	Wild type	[110]
Pectobacterium atrosepticum SCRI1043 Δ*rpoS*	Mutant strain of SCRI1043 containing the kanamycin cassette in the chromosome; Km^R	This study
Pectobacterium atrosepticum SCRI1043 Δ*rpoS* complementation mutant	Mutant strain of SCRI1043 containing the kanamycin cassette in the chromosome and the *rpoS* complementation construct on pGEM:*rpoS*; Km^R Amp^R	This study
Escherichia coli cc118	Host for suicidal vector pKNG101; *Δ(ara, leu) araD ΔlacX 74 galE galK PhoA20 thi-1 rpsE rpoB argE (am) recA1,* Sm^R	[111]
Escherichia coli HH26/pNJ5000	Mobilizing strain for conjugative transfer of the suicide vector pKNG101 into *P. atrosepticum* cells; *tra+;* Tet^R	[112]
Escherichia coli NovaBlue	*endA 1hsdR17 (rK12–mK12+) supE44 thi-1 recA1 gyrA96 relA1 lacF' [proA+ B+ lacIqZ ΔM15::Tn10 (*Tet^R*)]*	Novagen
	**Plasmids**	
pGEM-T Easy	Linearized vector for cloning; *f1 ori* Amp^R *lacZ*	Promega
pGEM:*rpoS*	*f1 ori* Amp^R *lacZ rpoS*	This study
pGEM:*ΔrpoS*;Km^R	*f1 ori* Amp^R *lacZ* Km^R	This study
pKD4	Matrix for PCR amplification of kanamycin resistance cassette; *oriRγ rgnB bla* Km^R	[113]
pKNG101	Suicide mobilized vector for inactivation of target genes; *pir-ori R6K mobRK2 sacB* Sm^R	[97]
pKNG101:*ΔrpoS*;Km^R	Suicide plasmid carrying mutant locus *ΔrpoS*;Km^R; Km^R Sm^R *sacB*	This study
pGEM:*rpoS*; complementation construct	*f1 ori* Amp^R *lacZ rpoS*	This study

	**Primers**
**Primer name**	**Primer sequence 5'─3'**
	**Primers for mutagenesis**
**up** *rpoS* F	TGGCAACAACGCTTCTGGTG
**dn** *rpoS* R	GAACTGAATGAAATCAAGGCGGAG

**dn** *rpoS* KmF	CCCATGTCAGCCGTTAAGACGCTGGAAGATGTGGGTCG
**up** *rpoS* KmR	CCAGCCTACACAATCGATGGGACATCGCCACGC
**Km** *rpoS* F	GCGATGTCCCATCGATTGTGTAGGCTGGAGCTGCTTC
**Km** *rpoS* R	ATCTTCCAGCGTCTTAACGGCTGACATGGGAATTAGC
**Check** *rpoS* F	CGTATAGCCTGAATGTCGGGCAATC
**Check** *rpoS* R	GCCATCAACTGGTTGCGTTGC
**T7F**	TAATACGACTCACTATAGGG
**Sp6R**	TAAATCCACTGTGATATCTTA
**Primers for complementation construct**	
**Comp** *rpoS* F	CGATACGATGCTAGTCCGTGAGC
**Comp** *rpoS* R	CTGGCGAATATCATACGCTAATCG
**Primers for qRT-PCR**	
*rpoS*_F	CTATTTGGGAGAGATTGGCTATTCGC
*rpoS*_R	CCAGACCACGATTGTTGTAACGGC
*rpoD*_F	CTAATGCGGAAGAAGACATTGCCCC
*rpoD*_R	CTTCTCACGCGCCAGTTCAGGATCG
*Ffh*_F	GTGGCATCATCCCTTTCATACCGC
*Ffh*_R	CGATTCAGGAACGTGCTAAACCAGAG
*recA*_F	GTGGATATTGATGCCTTCGCCG
*recA*_R	GTTTTATGCCTCTGTTCGTTTGGATATTC
*ECA0603_cfa6*_F	CGTGGTCGGGCATTCTGTAG
*ECA0603_cfa6*_R	CGGCGTAACATCTTCAACGG
*ECA0609_cfl*_F	CACAGACGAAATCGCATCTATATTTTAC
*ECA0609_cfl*_R	CTGGCTTATCACCATCTGATTTGTG
*ECA4106_pck*_F	CTCGGCAAACGTCGGCG
*ECA4106_pck*_R	CTTGATGGTTTTGGCGTAGCAG
*ECA3517_trxC*_F	CTTGGTGCGGTCCCTGCG
*ECA3517_trxC*_R	CGGTTCGGCTTCGGTATTGAC
*ECA1700_flgM*_F	CAATGTCCTGCGTGCCTGG
*ECA1700_flgM*_R	GCACACAGTCACTGAAGCCG
*ECA3238_iscR*_F	CTCATCCAGCGTGATGTTATTCAGG
*ECA3238_iscR*_R	CTGTTGGTATGGTTATTTCTGCCGTC
*ECA3882_dnaK*_F	CAGCACCAATGGCAACGG
*ECA3882_dnaK*_R	GAAACTGGAATCACTGGTAGAAGAGC
*ECA3014_treC*_F	GGTCTGTGGATTTTGGGCGG
*ECA3014_treC*_R	GGGCTGAAATACATCCTGACTGAAC
*ECA2112_hrpW*_F	CTGAGCACTGCTGTTAGTGATTTC
*ECA2112_hrpW*_R	CTGAAAAACGTGGTGTTTGG
*ECA2767_dps*_F	CTGGAACATGCGTGGCG
*ECA2767_dps*_R	CAGCGACGTTTGTTTACCCAC
*ECA2180_uvrD*_F	GCTTCAAGAGGAGATCTCTACATACAT
*ECA2180_uvrD*_R	GGAAGAGCGGCGTAAGTTTT
*ECA3430*_F	CTGGGCAAGACATACAGTGAAGGTAAG
*ECA3430*_R	CGCTCGGATGTAGCCTGC
*ECA3441*_F	GAAAGCGGCTAAAGCTGAGATTGCC
*ECA3441*_R	CATGAAGGGCAGCAAAATCGTC
*ECA4067_pelA*_F	CTGCTCGCAGCCCAACCG
*ECA4067_pelA*_R	GACGACTTTACCGCTTGAATC
*ECA4068_pelB*_F	CACGCCAGACAATGACACGAC
*ECA4068_pelB*_R	GCATGAACCAGACCACCACGC
